# The Association Between Bell’s Palsy and Vestibular Dysfunction in Relation to IgG Antibodies to Neurotropic Viruses

**DOI:** 10.3390/jcm14155290

**Published:** 2025-07-26

**Authors:** Krsto Dawidowsky, Srecko Branica, Lana Kovac Bilic, Zrinka Bosnjak, Marija Pastorcic-Grgic, Gorazd Poje, Barbara Dawidowsky

**Affiliations:** 1 Department of ENT&HNS, Zagreb University Hospital Center, School of Medicine, University of Zagreb, Kispaticeva 12, 10000 Zagreb, Croatia; 2 Department of Microbiology, Zagreb University Hospital Center, School of Medicine, University of Zagreb, 10000 Zagreb, Croatia; 3 Department of Ophthalmology, ENT & Neurosurgery, Children’s Hospital, School of Medicine, University of Zagreb, 10000 Zagreb, Croatia

**Keywords:** Bell’s palsy, vestibular impairment, serology against neurotropic viruses

## Abstract

**Background/Objectives:** The aetiology of Bell’s palsy remains unclear and is typically diagnosed by exclusion. This study investigated the potential role of neurotropic viruses and explored the relationship between facial nerve impairment and vestibular dysfunction to improve the understanding of the condition. **Methods:** Antibodies against herpes simplex virus (HSV) and varicella-zoster virus (VZV) were assessed using ELISA. Vestibular function was evaluated through computerised videonystagmography, rotatory chair, and clinical vestibulospinal assessments. Facial nerve lesion localisation was determined by stapedial reflex testing. Fisher’s exact test was used for statistical analysis. **Results:** Of 51 patients with Bell’s palsy, 62.7% exhibited vestibular dysfunction, and 70.6% were IgG-positive for at least one neurotropic virus. Vestibular impairment was significantly more common in seropositive patients. Statistically significant associations were observed between vestibular dysfunction and viral IgG seropositivity (*p* < 0.0001), the severity of vestibular dysfunction and facial paresis (*p* = 0.0126), and the side of vestibular impairment and the side of facial palsy (*p* < 0.0001), with 90.6% of cases showing ipsilateral involvement. **Conclusions**: These findings support the hypothesis that neurotropic viruses may act as a common pathological factor in both Bell’s palsy and associated vestibular dysfunction.

## 1. Introduction

Bell’s palsy is a peripheral facial nerve impairment of unknown origin. With an incidence of 15–40 cases per 100,000 individuals, it is the most common peripheral cranial nerve neuropathy. The clinical presentation of Bell’s palsy includes sudden unilateral paresis or paralysis of the entire facial musculature, occasionally accompanied by loss of salivation, lacrimation, and taste. Diagnosis is achieved by excluding all established causes of peripheral facial nerve damage, e.g., cases of trauma, infection, tumours, toxic, and iatrogenic lesions, as well as some congenital and neurological syndromes. Clinical expression varies from mild hemifacial weakness to complete facial paralysis, with reduced xerostomia, dysgeusia and xerophthalmia in some cases [1,2,3], and the degree of impairment is mostly determined by the House–Brackmann grading scale (HB) [4]. Symptoms usually last from three weeks to a couple of months, with a spontaneous recovery rate of almost 75%, even more in cases of mild palsy (HB 2 and 3). Therapy with high doses of corticosteroids given within the first 72 h of the onset of symptoms leads to complete recovery in more than 90% of Bell’s palsy patients. In patients with severe palsy (HB 4–6) and lagophthalmos, artificial tears (eye drops) with an eye patch/moist chamber should be used for eye protection [5,6,7,8]. Surgical therapy is required in rare cases that do not respond to conservative treatment [9].

The aetiology of Bell’s palsy continues to seek a definitive explanation even two centuries after the first description of this medical condition, despite extensive research efforts and diagnostic advances [10,11]. Studies exploring the pathogenesis of Bell’s palsy identified swelling of the facial nerve with consequent entrapment, as well as axonal degeneration in the labyrinth segment of the facial canal and around the geniculate ganglion [12,13,14,15,16]. The origin of this process remains unclear, with multiple aetiological theories proposed, including consideration of anatomical predisposition, environmental factors, ischemia, inflammation, immunology, and viral infection [17,18,19,20,21].

While no theory has been fully confirmed, many authors suggest that the focal reactivation of a latent neurotropic virus infection is the most probable cause. Most studies primarily focused on herpes virus type 1 and the varicella-zoster virus, and the implication of cytomegalovirus, Epstein–Barr virus, Usutu virus, herpes virus type 6, mumps, rubella, and human immunodeficiency virus has also been reported. The results did not conclusively identify a particular virus or process, leaving uncertainty whether reactivated viral inflammation or the virus-induced immune response caused intra-axonal degeneration.

The theory of the focal reactivation of neurotropic viruses proposes that Bell’s palsy is caused by the reactivation of latent viruses in the geniculate ganglion of the facial nerve. Neurotropic viruses (like HSV, VZV, EBV, CMV) establish lifelong latency in neural tissue, including the geniculate ganglion of the facial nerve. During latency, the virus remains dormant with no active replication. Stress, immunosuppression, or other unknown factors can lead to reactivation of the latent virus. Upon reactivation, the virus begins replicating and causes inflammation and swelling of the facial nerve within the narrow facial canal in the temporal bone, particularly in the area of the geniculate ganglion and labyrinthine segment, which was confirmed with MRI studies in Bell’s palsy. The inflammatory response and oedema compress the facial nerve disrupting axonal transport and blood flow, leading to facial muscle paralysis. Despite extensive research on viral reactivation mechanisms—including studies involving cerebrospinal fluid analysis, viral cultures, serology, facial nerve MRI, and PCR-based detection of viral DNA in saliva—reported positivity rates among Bell’s palsy patients range widely from 17% to 80%. While these findings suggest a potential viral aetiology, they have not conclusively established viral infection as the sole cause of Bell’s palsy. Moreover, serological studies have shown no significant differences in antibody titre against neurotropic viruses between patients with Bell’s palsy and healthy controls [20,21,22,23,24,25,26,27,28,29,30,31].

Among the main symptoms of Bell’s palsy, mild tinnitus and hearing and balance impairment have been observed in some patients. While significant hearing loss was not commonly reported in most studies, the results of vestibular testing in patients with Bell’s palsy varied, showing subjective and objective vestibular impairment in 5% up to 75% of the cases. This contrasts with the estimated incidence of vestibular dysfunction of 0.6% to 35% in the general population, suggesting higher incidence of vestibular impairment in Bell’s palsy patients [32,33,34,35]. A connection between neurotropic virus reactivation and vestibular dysfunction was also reported, with latent virus established in the vestibular (Scarpa’s) ganglion. Once reactivated, the virus can induce localised inflammation and vestibular nerve damage, presenting clinically with vertigo, nystagmus, and unsteadiness. Vestibular neuritis, a common cause of acute vertigo, is often linked to neurotropic virus reactivation (HSV, EBV, VZV) [36,37].

To the best of our knowledge, no previous study has specifically investigated the relationship between neurotropic viruses and vestibular function in patients with Bell’s palsy. Building on the theory that reactivation of latent viral infections may contribute to both Bell’s palsy and vestibular dysfunction, we conducted a prospective study to examine whether a shared viral aetiology could simultaneously affect the facial nerve and the vestibular system. As the facial nerve lesion in Bell’s palsy typically localises to the labyrinthine segment of the facial canal—an area anatomically adjacent to the vestibular apparatus—we focused on evaluating vestibular function in these patients. Given the phylogenetic and anatomical relationship between the two systems, we hypothesised that seropositive patients would exhibit some degree of vestibular dysfunction.

To explore this, we tested patients with Bell’s palsy for IgM and IgG antibodies against herpes simplex virus (HSV) and varicella-zoster virus (VZV), as these viruses are most frequently implicated in the literature as possible causative agents. We then analysed the association between vestibular dysfunction and the presence of viral IgG antibodies, expecting a higher incidence of vestibular impairment in seropositive individuals. Additionally, we examined whether the severity of facial nerve palsy was associated with the extent of vestibular dysfunction, and whether both impairments occurred on the same (ipsilateral) side.

## 2. Materials and Methods

Participants for this prospective study were chosen among all patients treated for peripheral facial paresis in our department over four years. In a total of 93 adult patients of both genders aged 19 to 74 years diagnosed with peripheral facial paresis, the cause of facial palsy was identified in 34 patients through clinical examination, on indication supplemented by MRI, audiovestibular, and laboratory testing. Their HB grade was determined before the treatment. Most of these patients presented with severe ear inflammation that had complicated into facial nerve palsy. They were treated with antibiotics and high-dose corticosteroids —prednisolone 60–80 mg daily for 5 days, followed by a tapering dose over the next 5–10 days. In cases showing clear clinical signs of viral infection, such as herpes simplex virus (HSV) or Ramsay Hunt syndrome, patients received antiviral therapy in addition to steroids (valacyclovir 1000 mg, three times daily for 7 days). Their blood was tested for the presence of the virus at the beginning of the treatment, and serological confirmation for the viral infection was confirmed in all cases after 2–6 days. We also identified four cases of cholesteatoma affecting the facial nerve. These patients were treated with corticosteroids and immediately underwent surgical intervention. Two patients had developed peripheral facial palsy within 12 to 24 h following head trauma. Both were treated with corticosteroids. Surgery was not required, as MRI confirmed that the palsy was due to facial nerve oedema, not structural damage. Additionally, one patient was diagnosed with Lyme borreliosis, presenting with facial paralysis after a tick bite, and was treated with ceftriaxone 2 g daily for 14 days. Importantly, in this cohort of 34 patients, no tumours of the temporal bone, internal auditory canal, or posterior fossa were found to be the cause of facial palsy. All 93 patients were prescribed facial muscle exercises, and in cases of lagophthalmos, appropriate eye protection measures were recommended.

The other 59 patients, aged 27 to 56 years, matched the criteria for newly occurring unilateral Bell’s palsy without a previous history of vestibular impairment and symptoms that lasted no more than 72 h. The patients were scored according to the House–Brackmann scale and treated immediately with prednisolone (as described earlier, with eye protection and facial exercises to prevent facial muscle atrophy). Their blood samples were tested for IgG and IgM antibodies against neurotropic viruses (HSV, VZV, CMV, EBV). Serological testing was conducted by ELISA method on the device BEP^®^ 2000 (Dade Behring Marburg GmbH; Marburg, Germany) which immediately reads the test results based on density or absorption using the appropriate software. EBNA IgG, VCA/EA IgG, and VCA IgM antibodies (VIDAS^®^ EBV, BioMerieux, bioMérieux SA, Marcy-l’Étoile, France were determined for EBV. For HSV types 1 and 2 IgM 1/2 and IgG 1/2 (NovagnostTM) antibodies, for VZV (Enzygnost^®^ Anti VZV), and for CMV (VIDAS^®^ CMV), IgM and IgG antibodies were determined. The tests for EBV and CMV were qualitative, with results reported as positive, marginal, or negative. In contrast, HSV and VZV were assessed using qualitative, quantitative or semiquantitative tests, with antibody titres expressed in U/mL. A result was considered positive if the titre exceeded 0.200 U/mL for VZV or 11.5 U/mL for HSV. Serum samples, in which a marginal or weakly positive result was obtained, were re-examined. In 8 of 59 patients, positive IgM and IgG titres for HSV, VZV, and EBV were found after 2–6 days, suggesting an acute viral infection. At the time of initial examination, these patients showed no clinical or laboratory signs of a viral disease. All presented with moderate facial palsy (House–Brackmann grades 2 to 4) as the only symptom and were initially classified as having Bell’s palsy. Upon receiving the serological results, they were treated with valacyclovir 1000 mg three times daily for 10 days, in addition to corticosteroids. All eight patients achieved complete recovery within 3 to 5 weeks. As acute viral inflammation was suspected as the cause of their facial paresis, these cases did not meet the strict criteria for idiopathic paresis (Bell’s palsy) and were therefore excluded from the study sample. This resulted in a final study group of 51 patients (27 female and 24 male) with Bell’s palsy (paresis of the facial nerve of unknown origin). Although antibodies to HSV, VZV, EBV, and CMV were measured, only HSV and VZV were included in the main analysis due to their stronger suspected link to Bell’s palsy. EBV and CMV were excluded because of their consistently high seroprevalence in the general population, which reduces diagnostic specificity and interpretability. Moreover, current reviews and meta-analyses consistently report limited evidence supporting the involvement of CMV or EBV in either Bell’s palsy or vestibular dysfunction.

In addition, 51 patients with Bell’s palsy underwent vestibular testing to detect possible dysfunction. Vestibular function was assessed using a computerised videonystagmography (CVNG) system (Hortman, GN, Ballerup, Denmark), which included evaluation of spontaneous and gaze-evoked nystagmus, saccadic eye movements, smooth pursuit, and bithermal caloric testing with water. Abnormalities in saccades and smooth pursuit, which may indicate central lesions (typically involving the brainstem or cerebellum), were analysed to help differentiate peripheral from central causes of dizziness or vertigo. The caloric test, performed with VNG goggles, assessed unilateral peripheral vestibular hypofunction. Jongkees’ formula was applied to calculate unilateral weakness (UW), vestibular sensitivity (VS), and directional preponderance (DP), with the latter aiding in the differentiation of peripheral dysfunction from central compensation or spontaneous nystagmus. These parameters were automatically derived by the CVNG system based on the recorded slow-phase velocity (SPV) values. A UW of 30% or greater, as determined by Jongkees’ formula, was considered pathological, indicating a ≥30% weaker response from one ear compared to the combined caloric responses of both ears, consistent with clinical standards. Patients underwent additional testing, including a rotatory chair test (evaluating gain, asymmetry, phase, and bias) and vestibulospinal clinical tests. Instability on the Romberg test or a sway >45° on the Fukuda–Unterberger stepping test was considered indicative of unilateral vestibular dysfunction. In this study, “vestibular dysfunction” specifically refers to unilateral weakness detected by caloric testing using CVNG and supported by findings from the rotatory chair and vestibulospinal clinical tests, which assess vestibular function at low to mid frequencies. High-frequency vestibular function was not assessed, as video head impulse testing (vHIT) was not performed. The location of the facial nerve lesion within the canal was determined by stapedial reflex (STAR) testing on the AT235h tympanometer (Ineracoustics, Middelfart, Denmark). Absence of the reflex indicates a nerve lesion proximally, above the separation of the branch for the stapedial muscle. Hearing thresholds were assessed using pure-tone audiometry with the AC 40 audiometer (Interacoustics, Denmark). Only newly detected sensorineural hearing loss (occurring concurrently with the onset of Bell’s palsy) was considered pathological.

To analyse the relationship between Bell’s palsy and vestibular dysfunction in the context of neurotropic viral presence, we categorised patients by the severity of facial nerve paresis and the severity of vestibular dysfunction. Statistical analysis was performed using IBM SPSS Statistics 24.0 (IBM Corp., Armonk, NY, USA). For comparison of variables, Fisher’s exact test was used.

## 3. Results

**Figure 1:**
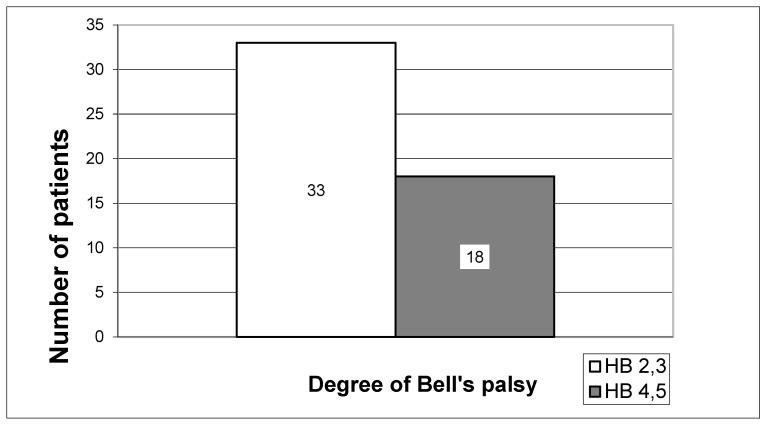
Distribution of patients according to the degree of Bell’s palsy using the House–Brackmann scale.

The severity of the Bell’s palsy was determined according to the House–Brackmann grading scale (HB). It is a standard six-stage method for subjectively assessing facial nerve paresis, evaluating muscle function at rest and during movement, as well as secondary defects such as hemifacial spasm or synkinesis. The distribution of 51 patients in 5 groups according to the original HB gradients would have led to dispersion of the results, with the groups being too small for reliable statistical processing. Hence, two groups were formed: the first included 33 patients with mild (HB2 and HB3), and the second 18 patients with severe paresis (HB4 and HB5). None of our patients had paralysis (HB6) (Figure 1).

Hearing levels were assessed using pure-tone audiometry (PTA), and the presence of the stapedial reflex (STAR) was evaluated with a tympanometer. No new cases of sensorineural hearing loss were identified, and among patients with previous audiograms, no significant changes in hearing thresholds were observed. Tympanometry results were predominantly normal (type A curves) in most patients. However, three patients exhibited type B and five showed type C curves, indicating possible eustachian tube dysfunction. These patients were additionally treated with nasal corticosteroids for 6 weeks and decongestants for 7 days. Tympanometric testing of the stapedial reflex (STAR) showed negative reflex on the side of Bell’s palsy in all subjects, indicating suprastrapedial lesion of the facial nerve.

**Figure 2:**
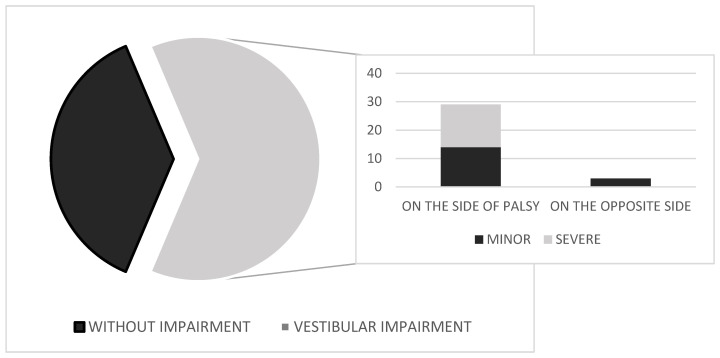
Vestibular impairment in the group of patients with Bell’s palsy.

The results of vestibular testing—based on abnormalities identified using CVNG, rotatory chair, and clinical vestibulospinal tests, reflecting dysfunction at low to mid frequencies—are shown in Figure 2. In a group of 51 patients with Bell’s palsy, unilateral vestibular weakness was found in 32 subjects (62.7%). All patients included in the study had no prior history or documented record of vestibular dysfunction. Caloric testing in CVNG showed unilateral peripheral impairment in all 32 cases, with horizontal, unidirectional nystagmus beating away from the affected (weaker) ear. The nystagmus was fatigable and suppressed with fixation. In mild cases, it was first-degree per Alexander’s classification; in more pronounced cases, first- and second-degree. No abnormalities were observed in saccadic or smooth pursuit eye-movement testing on CVNG. Of 32 patients, 29 had vestibular impairment on the side of the Bell’s palsy, while 3 had impairment on the opposite side. The intensity of vestibular dysfunction was assessed in subjects with abnormal vestibular test results using CVNG and further evaluated with the rotatory chair test and clinical vestibulospinal tests, especially in cases where caloric testing with CVNG was borderline. Of the 32 patients, 18 had more pronounced impairment (UW 41–50%) and 14 had mild vestibular dysfunction (UW 30–40%). None of the 14 patients in the group with minor vestibular dysfunction presented clinically with vertigo, nausea, or vomiting. Only four of them reported experiencing mild dizziness. All these patients underwent additional testing with the rotatory chair test and clinical vestibulospinal assessments (Romberg and Fukuda–Unterberger tests), to confirm peripheral vestibular dysfunction. In the group of 18 patients with more pronounced dysfunction, eight exhibited clear clinical symptoms of moderate vertigo, while the remaining patients reported dizziness and gait instability. Among those with vertigo, only five experienced nausea, and none reported vomiting. CVNG, rotatory chair testing, and vestibulospinal assessments revealed more pronounced unilateral peripheral weakness, ranging from 41% to 50%.

**Figure 3:**
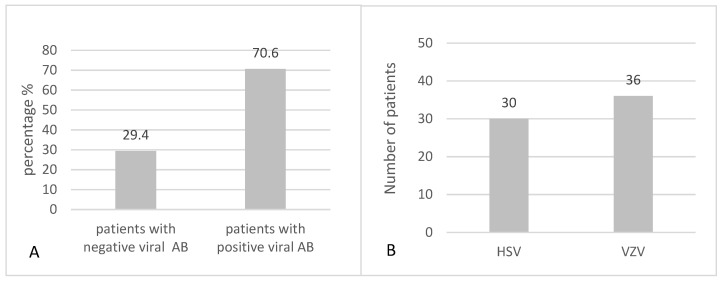
(**A**) Viral antibodies in patients with Bell’s palsy. (**B**) Distribution of antibodies by type of virus in patients with Bell’s palsy.

IgG antibody titres for HSV and VZV were evaluated in serum samples from 51 patients with Bell’s palsy using the ELISA method. Of these, 36 patients (70.6%) were seropositive for one or both viruses (Figure 3A), and the virus-specific distribution is shown in Figure 3B. Quantitative results showed that 30 patients (58.8%) were positive for HSV IgG (>11.5 U/mL) and 36 (70.6%) for VZV IgG (>0.200 U/mL). These results reflect prior exposure and potential for viral reactivation. Our analysis focused on overall seropositivity to neurotropic viruses rather than on a specific pathogen, as no single virus has been definitively confirmed as the cause of Bell’s palsy in previous studies. Moreover, because 80.6% of seropositive patients (29 of 36) had antibodies to both HSV and VZV, separate virus-specific analyses were not feasible. This approach allowed us to explore broader associations between viral exposure and vestibular involvement.

Furthermore, we performed a statistical analysis to test the hypothesis that vestibular impairment in Bell’s palsy may be associated with neurotropic viral involvement. The relationship between vestibular impairment and viral antibodies in patients with Bell’s palsy was analysed with Fisher’s exact test. The patients were grouped according to the presence of viral IgG antibodies in serum and the presence of vestibular dysfunction (Table 1). Among the 51 patients with Bell’s palsy, 36 (70.6%) were IgG-positive for either HSV or VZV. Of these seropositive patients, 30 (83.3%) exhibited vestibular dysfunction. In contrast, only 2 of the 15 seronegative patients (13.3%) had vestibular impairment. The difference was statistically significant (Fisher’s exact test, *p* < 0.0001), suggesting an association between viral seropositivity and the presence of vestibular dysfunction in Bell’s palsy patients.

To examine whether unilateral vestibular dysfunction tends to occur on the same side as Bell’s palsy, we compared patients with same-sided vestibular impairment to those with no or contralateral dysfunction (Table 2). Vestibular dysfunction was significantly more frequent on the side affected by facial palsy: of 32 patients with vestibular impairment, 29 (90.6%) had dysfunction on the same side as Bell’s palsy, while only 3 (9.4%) showed contralateral involvement. In contrast, most patients without vestibular dysfunction (68.6%) had normal findings on the side opposite the facial palsy, supporting the link between facial palsy and same-sided vestibular involvement. The difference was statistically significant (Fisher’s exact test, *p* < 0.0001), supporting a potential localised mechanism linking facial and vestibular nerve involvement.

We also tested the relation of the degree of facial paresis with the severity of vestibular dysfunction. Among the 32 patients with confirmed vestibular impairment, 16 had mild Bell’s palsy (HB grade 2–3) and 16 had more severe paresis (HB grade 4–5). Each group was then assessed for the severity of vestibular dysfunction. In the mild paresis group, 11 out of 16 patients (68.8%) exhibited mild vestibular dysfunction (unilateral weakness, UW, 30–40%). In contrast, in the severe paresis group, 13 out of 16 patients (81.3%) showed more pronounced vestibular dysfunction (UW 41–50%) (Table 3). This difference was statistically significant (Fisher’s exact test, *p* = 0.0126), suggesting a possible association between the extent of facial nerve involvement and the severity of vestibular impairment.

A summary of the main associations is provided in Table 4. Patients with vestibular dysfunction showed significantly higher IgG seropositivity to HSV and/or VZV, more frequently had facial palsy on the same side as the vestibular impairment, and more often exhibited severe paresis (HB4–HB5). These findings support the hypothesis of a potential link between viral exposure and combined facial and vestibular involvement. Although antibodies to CMV and EBV were measured, these viruses were not included in the main analysis due to their high background seroprevalence in the general population and limited clinical relevance in Bell’s palsy, as suggested by previous studies.

## 4. Discussion

Although direct evidence remains limited, the theory of focal latent reactivation of neurotropic viruses is widely considered the most probable cause of Bell’s palsy [38,39,40,41,42]. Some authors have extended this hypothesis beyond the facial nerve, implicating viral reactivation in vestibular and other cranial nerve disorders. Anatomical studies have shown that the facial nerve is most frequently affected in the labyrinthine segment and geniculate ganglion [43,44,45,46,47]. Given that the facial and vestibular nerves emerge from the cerebellopontine angle, share the internal auditory canal, and develop in parallel from the branchial arch system, we hypothesised that both may be susceptible to the same localised pathological process—namely, viral reactivation. To evaluate the plausibility of a shared pathological mechanism, we examined whether vestibular dysfunction in Bell’s palsy patients is associated with viral antibody presence, laterality of nerve involvement, and the relative severity of both conditions. Unlike previous studies that compared Bell’s palsy patients to healthy controls, our analysis focused on associations within a clinically defined group—that is, patients diagnosed with Bell’s palsy, some with and some without vestibular dysfunction, and with differing serological results for neurotropic viruses. This design was selected given the inconsistency of prior findings on seroprevalence and vestibular impairment in comparisons with healthy populations.

We included 51 patients with newly occurred Bell’s palsy (within 72 h of symptom onset), all of whom had no prior vestibular complaints. Facial nerve function was graded using the House–Brackmann scale: patients were categorised as having mild (HB2–HB3) or severe (HB4–HB5) paresis, with none presenting with complete paralysis (HB6). All had a negative stapedial reflex on the affected side, indicating a suprastrapedial lesion, in line with previous anatomical findings [48,49,50].

Vestibular dysfunction was observed in 62.7% of patients, which aligns with the wide prevalence range (5–75%) reported in previous studies [32,33,34,35]. In our cohort, 32 out of 51 patients (62.7%) demonstrated unilateral weakness on caloric testing, indicating low-frequency vestibular hypofunction. These findings were further supported by abnormalities in the rotatory chair test, which assesses vestibular responses at low to mid frequencies. However, high-frequency vestibular function—typically assessed using the video head impulse test (vHIT)—was not evaluated in our study, so the results may not fully reflect dysfunction across the entire frequency spectrum. Although advanced tests such as vHIT and vestibular-evoked myogenic potentials (VEMPs) were not included, our diagnostic protocol—comprising videonystagmography (VNG), rotatory chair testing, and clinical vestibulospinal assessments—was sufficient to detect general vestibular dysfunction. This approach aligned with the aim of our study: to identify vestibular involvement and explore its potential association with neurotropic viruses (HSV and VZV), rather than to establish specific vestibular disorders. Of the 32 affected patients, 14 had mild impairment (30–40% unilateral weakness), confirmed by additional vestibular tests, while 18 exhibited more pronounced dysfunction (41–50% UW). No cases of severe dysfunction (UW > 50%) were observed. In the group of patients with more pronounced vestibular impairment, only eight reported moderate vertigo, while the others complained of dizziness and instability. All received corticosteroids, along with the rest of the cohort, and vestibular suppressants (dimenhydrinate 60 mg daily for 10 days) and recovered fully within 3–6 weeks. Follow-up CVNG testing showed resolution of vestibular signs in all cases. Further neurological and imaging evaluations in those eight patients (MRI, angiography, Doppler) excluded other central or structural causes.

Previous studies have shown that 29–90% of patients with Bell’s palsy are IgG-positive for neurotropic viruses, reflecting widespread exposure in the general population [51,52,53,54,55,56]. As no single virus has been conclusively identified as the causative agent, we focused on overall seropositivity, interpreted as a marker of possible reactivation. Obtaining direct evidence of viral replication during the acute phase of facial nerve involvement is exceptionally challenging. Invasive sampling of the nerve is not possible in living patients, and post-mortem studies are both limited and inconsistent. Moreover, while viral DNA has been detected in some cases, it has also been found in asymptomatic individuals. In this context, we aimed to explore patterns of immune response rather than confirm a specific pathogen.

The phylogenetic, anatomical, and embryological relationship between the facial and vestibulocochlear nerves may help explain their concurrent involvement in Bell’s palsy, particularly given the propensity of neurotropic viruses to reactivate locally and affect multiple closely situated neural structures [25,26]. Reactivation of HSV-1 or VZV in the geniculate ganglion may lead to localised inflammation, selectively affecting adjacent nerve branches/vestibular structures depending on viral activity and host factors [24]. Sparing of the cochlear nerve and hearing may be due to its distinct vascular supply and fascicular organisation, making it less vulnerable to localised inflammatory damage [27]. The proposed mechanism involving viral reactivation of both facial and vestibular nerves is most plausibly linked to HSV-1, having been implicated in both Bell’s palsy and vestibular neuritis through involvement of the geniculate and superior vestibular nerves. VZV, by contrast, is more commonly linked to Ramsay Hunt syndrome, which frequently involves cochlear symptoms and broader cranial nerve damage. In our study, only patients with elevated IgG titres—indicating prior infection and potential for neurotropic viral reactivation—were included. Herpes simplex virus (HSV) and varicella-zoster virus (VZV) were selected for analysis due to their frequent implication in Bell’s palsy across previous studies. Although cytomegalovirus (CMV) and Epstein–Barr virus (EBV) were also tested (with 76.4% and 92.2% seropositivity, respectively), they were excluded from the main analysis owing to their high prevalence in the general population (e.g., >90% for EBV), which reduces interpretive value [24,30,31]. Quantitative IgG titres were available for HSV and VZV, with 30 patients (58.8%) testing positive for HSV and 36 (70.6%) for VZV. Overall, 70.6% of patients were seropositive for at least one of the tested neurotropic viruses, most commonly for both. Due to this high overlap, separate virus-specific analysis was not feasible, and the focus remained on overall seropositivity as a marker of potential reactivation. Patients with positive IgM titres were excluded to avoid confounding by acute infection. A healthy control group was not included, as our primary aim was to investigate associations within the Bell’s palsy population. However, future studies including control groups with more detailed analysis of IgM and IgG levels would be valuable for distinguishing disease-specific patterns from background viral exposure.

To date, only one cadaveric study has investigated viral involvement of both the facial and vestibular nerves [57], while clinical evidence remains limited. Vestibular impairment and a high prevalence of viral antibodies are found both in the general population and among patients with Bell’s palsy. Although the incidence of these findings was relatively high in our cohort, it aligns with previously reported data. Age-related effects on vestibular dysfunction were not assessed, as the sample size was insufficient to support subgroup analysis without introducing statistical overdispersion.

To the best of our knowledge, this is the first study to examine the relationship between vestibular dysfunction and viral antibodies in patients with Bell’s palsy, revealing that 58.8% of patients had both vestibular impairment and positive IgG titres. Statistical analysis confirmed an association between vestibular dysfunction and viral seropositivity in Bell’s palsy patients (*p* < 0.0001). We also observed a strong association between the side of facial palsy and the side of vestibular impairment (*p* < 0.0001): 90.6% of patients with vestibular dysfunction had it on the same side as their facial palsy. Three patients exhibited contralateral vestibular impairment (a frequency consistent with incidental findings in the general population). However, given the high overall rate of viral seropositivity, focal viral reactivation remains a possible explanation for these lesions as well [56].

A statistically significant association was also found between the severity of facial palsy and the degree of vestibular dysfunction (*p* = 0.0126). Among patients with more severe paresis (HB4-HB5), 81.3% showed pronounced vestibular impairment, whereas 68.8% of those with milder paresis (HB2-HB3) exhibited only mild dysfunction. These findings are in line with some previous reports suggesting that nystagmus in Bell’s palsy patients tends to resolve more rapidly when facial nerve involvement is less severe, although other studies have reported mixed results regarding this association [58,59,60,61,62].

## 5. Conclusions

In conclusion, our findings suggest a possible association between vestibular dysfunction—assessed through CVNG, rotatory chair, and clinical vestibulospinal tests—and prior exposure to neurotropic viruses (HSV and VZV) in patients with Bell’s palsy. Vestibular impairment was frequently observed on the same side as facial palsy and tended to be more pronounced in patients with more severe paresis. Seropositive patients were more likely to exhibit vestibular dysfunction, supporting the hypothesis of a potential shared pathological mechanism. However, a direct causal relationship between viral exposure and nerve impairment could not be established.

Clinically, the results highlight the potential value of including vestibular assessment in the evaluation of Bell’s palsy, particularly in patients with more severe symptoms or positive viral serology. The early detection of vestibular involvement may be relevant for clinical decision-making and rehabilitation. Nonetheless, given the observational design, small sample size (especially in the virus-negative subgroup), and lack of longitudinal data, the conclusions should be interpreted with caution. Additionally, since our vestibular testing primarily assessed low- to mid-frequency function, the absence of high-frequency assessments (e.g., vHIT) may limit the full characterisation of vestibular involvement. Future studies involving larger cohorts (including HB6 patients), control groups, and combined virological and vestibular testing (e.g., PCR, serology, CVNG, vHIT, VEMPs, MRI) are needed to explore these associations further and better understand the underlying mechanisms.

## Figures and Tables

**Table 1:** **Association between viral antibody presence and vestibular dysfunction in Bell’s palsy patients**. Patients with Bell’s palsy who were IgG-positive for neurotropic viruses showed a significantly higher incidence of vestibular dysfunction compared to virus-negative patients. Fisher’s exact test, *p* < 0. 0001.

	IgG VIRAL ANTIBODIES		TOTAL
	POSITIVE (*n* = 36)	NEGATIVE (*n* = 15)	
VESTIBULAR DYSFUNCTION (*n* = 32)	**30** (83.3%)	2 (13.3%)	32 (62.7%)
NO VESTIBULAR DYSFUNCTION (*n* = 19)	6 (16.7%)	**13** (86.7%)	19 (37.3%)
TOTAL	36 (100%)	15 (100%)	51

Fisher’s exact test *p* < 0.0001.

**Table 2:** **Laterality of vestibular dysfunction relative to the side of Bell’s palsy.** Vestibular impairment occurred predominantly on the same side as the facial nerve palsy, suggesting a localised effect. Fisher’s exact test, *p* < 0.0001.

	BELL’S PALSY		TOTAL
	AFFECTED SIDE (*n* = 51)	HEALTHY SIDE (*n* = 51)	
VESTIBULAR DYSFUNCTION (*n* = 32)	**29** (90.6%)	3 (9.4%)	32 (100%)
NO VESTIBULAR DYSFUNCTION (*n* = 70)	22 (31.4%)	**48** (68.6%)	70 (100%)
TOTAL	51	51	102

Fisher’s exact test *p* < 0.0001.

**Table 3:** **Relationship between Bell’s palsy severity and degree of vestibular dysfunction.** Patients with more severe facial paralysis (HB4–HB5) more frequently exhibited pronounced vestibular impairment, indicating a possible link between the extent of nerve involvement and vestibular function. Fisher’s exact test, *p* = 0.0126.

	VESTIBULAR DYSFUNCTION		TOTAL
	MILD (*n* = 14)	MORE PRONOUNCED (*n* = 18)	
WEAK BELL’S PALSY (HB2, HB3)	**11** (68.8%)	5 (31.2%)	16
HEAVY BELL’S PALSY (HB4, HB5)	3 (18.7%)	**13** (81.3%)	16
TOTAL	14	18	32

Fisher’s exact test *p* = 0.0126.

**Table 4:** Summary table showing associations between vestibular dysfunction and key clinical, serological, and neurological variables in patients with Bell’s palsy. HSV and VZV IgG seropositivity were combined due to high co-occurrence and similar pathogenic mechanisms. Facial paresis severity is categorised using House–Brackmann (HB) grades 2–3 (mild) and 4–5 (moderate to severe). Vestibular dysfunction was assessed using caloric testing and supported by rotatory chair and vestibulospinal tests. CMV and EBV were not included in the main analysis due to their high seroprevalence and limited evidence of involvement in Bell’s palsy or vestibular dysfunction.

Dependent Variable	Vestibular Dysfunction		Total (N = 51)	Statistical Significance
	YES	NO		
IgG seropositivity for HSV and/or VZV	30 (83.3%)	6 (16.7%)	36	*p* < 0.0001 (Fisher)
Bell’s palsy side vs. Vestibular dysfunction (same side)	29 (90.6%)	3 (9.4%)	32	*p* < 0.0001 (Fisher)
Facial paresis HB 2–3 with mild vestibular dysfunction	11 (68.8%)	5 (31.2%)	16	*p* = 0.0126 (Fisher)
Facial paresis HB 4–5 with more pronounced vestibular dysfunction	13 (81.3%)	3 (18.7%)	16	*p* = 0.0126 (Fisher)

## Data Availability

All data generated or analysed during this study are included in this article. Further enquiries can be directed to the corresponding author.

## Abbreviations

The following abbreviations are used in this manuscript:

EBV	Epstein–Barr virus
HSV	Herpes virus
CMV	Cytomegalovirus
VZV	Varicella-zoster virus
IgG	Immunoglobulin G
HB	House–Brackmann grading scale
UW	Unilateral vestibular weakness
